# Identification of septoria nodorum blotch susceptibility genes in hard winter wheat

**DOI:** 10.1007/s00122-026-05359-7

**Published:** 2026-09-15

**Authors:** Anju Maan Ara, Danielle J. Holmes, Timothy L. Friesen, Brett F. Carver, Guihua Bai, Paul St. Amand, Amy Bernado, Rajat Sharma, Meriem Aoun

**Affiliations:** 1 Department of Entomology and Plant Pathology, Oklahoma State University, Stillwater, OK USA; 2 Cereal Crops Improvement Research Unit, USDA-ARS, Fargo, ND USA; 3 Department of Plant and Soil Sciences, Oklahoma State University, Stillwater, OK USA; 4 Department of Agronomy, Kansas State University, Manhattan, KS USA; 5 Hard Winter Wheat Genetics Research Unit, USDA-ARS, Manhattan, KS USA

## Abstract

**Key message:**

Characterized and unknown septoria nodorum blotch sensitivity/resistance genes were identified in contemporary US hard winter wheat.

**Abstract:**

The necrotrophic fungus *Parastagonospora nodorum* is the causal agent of septoria nodorum blotch (SNB) of wheat. To determine the prevalence of SNB sensitivity genes in a contemporary US hard winter wheat (HWW), we evaluated a panel of 619 breeding lines and cultivars against five *P. nodorum* isolates and five necrotrophic effectors (NEs), SnToxA, SnTox1, SnTox3, SnTox267, and SnTox5, and genotyped the panel using genotyping-by-sequencing (GBS) markers and diagnostic kompetetive allele-specific PCR (KASP) markers for the sensitivity genes *Tsn1-B1*, *Snn1-B1*, and *Snn3-B1/B2*. GBS analysis identified 34,357 GBS-single-nucleotide polymorphism (SNP) markers*.* Evaluations against *P. nodorum* isolates showed that 40–67% of the genotypes were susceptible in the panel. Toxin infiltration assays showed that 54%, 2%, 37%, 13%, and 15% of the genotypes were sensitive to SnToxA, SnTox1, SnTox3, SnTox267, and SnTox5, respectively. Diagnostic KASP markers for *Tsn1-B1*, *Snn1-B1*, and *Snn3-B1/B2* showed prediction accuracies of 98%, 75%, and 92% for the corresponding effectors SnToxA, SnTox1, and SnTox3, respectively. Genome-wide association studies (GWAS) not only confirmed the presence of the previously characterized sensitivity genes *Tsn1-B1*, *Snn1-B1*, *Snn2*, *Snn3-B1/B2*, and *Snn5-B1*, but also identified new loci to be associated with responses to *P. nodorum* isolates and NEs. Of these, *Qsnb.osu-2AS* on chromosome 2AS was associated with responses to all five isolates. KASP markers linked to *Snn5-B1*, *Snn2*, and *Qsnb.osu-2AS* were developed for potential use in marker-assisted selection. These findings should guide breeding for SNB resistance in hard winter wheat.

**Supplementary Information:**

The online version contains supplementary material available at https://doi.org/10.1007/s00122-026-05359-7.

## Introduction

Common wheat (*Triticum aestivum* L., 2n = 6x = 42, AABBDD) is cultivated on more than 215 million hectares globally (Singh et al. 2022) and is one of the most important food crops. Hard winter wheat (HWW) is the most produced market class in the USA and is mostly cultivated in the Great Plains. However, wheat production is reduced due to multiple abiotic and biotic stresses. Septoria nodorum blotch (SNB), caused by the necrotrophic fungus, *Parastagonospora nodorum* (Berk.) (syn. *Septoria nodorum*, *Stagonospora nodorum*; teleomorph *Phaeosphaeria*), has become an economically important disease over the past several decades in many wheat-growing areas worldwide, including the eastern US, parts of northern Europe, Australia, and parts of North Asia (Bhathal et al. 2003; Murray and Brennan 2009; Crook et al. 2012; Figueroa et al. 2017; Cowger et al. 2020). *Parastagonospora nodorum* infects both leaves, causing leaf blotch, and spikes, causing glume blotch. This disease can reduce yield by up to 50% on susceptible cultivars (Liu et al. 2004b). In the Great Plains, SNB has become more prevalent in recent years (Aoun and Carver 2024). Farming practices such as minimum or no tillage that leave more crop residue on the soil surface could have contributed to the increased prevalence of SNB. Furthermore, a study of *P. nodorum* isolates collected from multiple wheat-growing regions in the US showed that isolates collected from HWW in Oklahoma had higher genetic and virulence diversity (Richards et al. 2019). This diversity may have contributed to adaptation to HWW cultivars grown in Oklahoma.

Genetic dissection of wheat−*P. nodorum* pathosystem has revealed that the recognition of *P. nodorum* necrotrophic effectors (NEs) by host sensitivity genes in an inverse gene-for-gene manner leads to programed cell death in the host and consequently wheat susceptibility (Friesen and Faris 2021; Peters Haugrud et al. 2022; Kariyawasam et al. 2023). To date, seven wheat sensitivity genes (*Tsn1-B1*, *Snn1-B1*, *Snn2*, *Snn3-B1*, *Snn3-B2*, *Snn3-D1*, and *Snn5-B1*) (Running 2022; Seneviratne et al. 2024a, b; Zhang et al. 2025; Running et al. 2025) and five *P. nodorum* NE genes (*SnToxA*, *SnTox1*, *SnTox3*, *SnTox5*, and *SnTox267*) have been cloned (Ciuffetti et al. 1997; Liu et al. 2004a, 2012; Friesen et al. 2007, 2008, 2012; Gao et al. 2015; Shi et al. 2015a). Thirteen sensitivity wheat gene–NE interactions have been identified, including *Tsn1-B1*–*SnToxA* (Friesen et al. 2006, 2009; Zhang et al. 2009; Faris and Friesen 2009; Faris et al. 2010, 2011; Running et al. 2025), *Tsn1-B2*–*SnToxA* (Running et al. 2025), *Snn1-B1*–*SnTox1* (Liu et al. 2004a, b; Reddy et al. 2008; Liu et al. 2012; Shi et al. 2016a; Seneviratne et al. 2024a), *Snn1-B2*–*SnTox1* (Seneviratne et al. 2024a), *Snn2*–*SnTox267* (Friesen et al. 2007, 2009; Zhang et al. 2009; Richards et al. 2021), *Snn3-B1*–*SnTox3* (Friesen et al. 2008; Liu et al. 2009; Shi et al. 2016b; Zhang et al. 2025), *Snn3-B2*–*SnTox3* (Zhang et al. 2025), *Snn3-D1*–*SnTox3* (Friesen et al. 2008; Liu et al. 2009; Zhang et al. 2011), *Snn4*–*SnTox4* (Abeysekara et al. 2009, 2012), *Snn5*-*B1*–*SnTox5* (Friesen et al. 2012; Sharma et al. 2019; Running 2022; Kariyawasam et al. 2022), *Snn5*-*B2*–*SnTox5* (Running 2022), *Snn6*–*SnTox267* (Gao et al. 2015; Richards et al. 2022), and *Snn7*–*SnTox267* (Shi et al. 2015; Richards et al. 2022)*.* Other studies showed that wheat–*P. nodorum* interactions are not necessarily defined by sensitivity gene–NE interactions and that resistance to SNB can be controlled by multiple quantitative trait loci (QTL) and affected by genotype-by-environment (G × E) interactions (Francki 2013, 2021; Cowger et al. 2020; Downie et al. 2021). Furthermore, seedling and adult plant SNB resistance may be controlled by different genes (Tommasini et al. 2007; Shankar et al. 2008), and response to leaf blotch and glume blotch can also be independently inherited (Francki 2013; Francki et al. 2018; Cowger et al. 2020).

Previous studies identified SNB sensitivity genes and resistance loci and their prevalence in various wheat classes (Phan et al. 2018; Ruud et al. 2019; Cowger et al. 2020; Peters Haugrud et al. 2023; Szabo-Hever et al. 2025a); however, data specific to contemporary HWW grown in the US Great Plains are limited. It has been reported that differences in *P*. *nodorum* sensitivity genes between wheat germplasm do exist (Crook et al. 2012; Richards et al. 2019). Given the rising incidence of SNB in the HWW production regions, elucidating the genetic basis of resistance/susceptibility in HWW is essential to guide effective selection within breeding programs. In this study, we investigated the genetics of susceptibility/resistance to SNB in a panel of 619 HWW genotypes, including elite breeding lines developed by the Oklahoma State University (OSU) wheat breeding program, along with cultivars from both the OSU and Kansas State University (KSU) breeding programs. We evaluated the panel at the seedling stage against *P. nodorum* isolates and NEs. Furthermore, we determined the prevalence of the sensitivity genes *Tsn1-B1*, *Snn1-B1*, *Snn3-B1*, and *Snn3-B2* in the panel using available diagnostic kompetitive allele-specific PCR (KASP) markers (Seneviratne et al. 2024a; Zhang et al. 2025; Running et al. 2025). To identify genomic regions associated with SNB susceptibility/resistance in this panel, a genome-wide association study (GWAS) was conducted using the phenotypic data, single-nucleotide polymorphism (SNP) generated via genotyping by sequencing (GBS), and diagnostic KASP markers available for currently characterized sensitivity genes. GWAS-significant SNPs associated with novel resistance/susceptibility loci, as well as characterized sensitivity genes that currently lack available diagnostic markers, were targeted for the development of KASP markers.

## Materials and methods

### Plant materials and genotyping

A panel of 619 elite HWW breeding lines and cultivars was used in this study. This panel includes 532 doubled-haploid breeding lines selected from 14 bi-parental crosses and 38 OSU elite breeding lines from the OSU wheat breeding program, and 49 cultivars from the OSU and KSU wheat breeding programs (Supplementary Table S1). This panel was genotyped using GBS (Poland and Rife 2012) at the USDA-ARS Central Small Grain Genotyping Lab in Manhattan, KS. SNP genotype calling was performed using TASSEL software v.5 (Bradbury et al. 2007), and the physical positions of SNPs were assigned using the Chinese Spring RefSeqv2.1 developed by the International Wheat Genome Consortium (IWGSC) (Zhu et al. 2021). SNP markers with missing data ≥ 60% were excluded, and missing data were imputed using Beagle 5 (Browning et al. 2018). Markers with minor allele frequency (MAF) ≤ 5% and heterozygosity ≥ 15% were removed from further analysis, which left 34,357 SNP markers (Supplementary Table S2) for downstream analyses.

Additionally, the HWW panel was genotyped using diagnostic KASP markers available for three previously characterized sensitivity genes *Tsn1* (*Tsn1-B1_1Ka* and *Tsn1-B1_2Ka*), *Snn1* (*Snn1_null*), and *Snn3* (*Snn3-B1* and *Snn3-B2*) (Seneviratne et al. 2024a; Zhang et al. 2025; and Running et al. 2025) (Supplementary Fig. S1). Genomic DNA was extracted from leaf tissue collected at the second leaf stage using a modified cetyl-trimethyl-ammonium-bromide (CTAB) method (Riede and Anderson 1996; Liu et al. 2006). DNA concentration was quantified using a Nanodrop spectrophotometer ND-8000 (ThermoFisher Scientific, Waltham, MA), and DNA stocks were diluted to a working concentration of 30 ng µL^−1^. KASP assay was performed following the modified LGC bioscience technologies thermal cycling protocol (Makhoul et al. 2020). Each 100 µL primer mix contained 12 µL of each allele-specific primer (100 µM), 30 µL of the common primer (100 µM), and 46 µL of ddH_2_O. Each PCR reaction consisted of 5 µL of 2 × KASP master mix (KBS-1050-102; LGC Biosearch Technologies Hoddesdon, UK), 0.14 µL primer mix, 1.86 µL of ddH_2_O, and 3 µL of DNA (30 ng/µL). The PCR was conducted on a Bio-Rad CFX-96 Opus real-time PCR system (Bio-Rad, Hercules, CA) with 94 °C for 15 min, followed by 10 touchdown cycles of denaturation at 94 °C for 20 s, annealing–extension starting at 65 °C for 1 min with a decrement of 0.8 °C at each cycle, followed by 30 cycles of denaturation at 94 °C for 20 s, and annealing–extension at 57 °C for 1 min. The fluorescent signal was measured after cooling down to 37 °C. Endpoint fluorescence reading and genotype clustering were performed in Bio-Rad CFX Maestro Software 2.3. Up to three additional cycles of 94 °C for 20 s and 57 °C for 1 min were added to the previous protocol if the clusters were not well separated after initial PCR.

### Seedling evaluation using *P. nodorum* isolates

*Parastagonospora nodorum* isolates OKG16-Sn1, OKG16-Sn2, OKG16-Sn9, OKG16-Sn13, and OKG16-Sn16 used in this study were collected from the winter wheat cultivar ‘Gallagher’ in Canadian County, Oklahoma in 2016 (Richards et al. 2019). These isolates were described based on their responses to the differential set of wheat lines (Supplementary Table S3) and the presence/absence of effectors SnToxA, SnTox1, SnTox3, SnTox267, and SnTox5 (Table 1). The isolates were grown on V8-potato dextrose agar (PDA) (150 ml of V8 juice, 3 g of CaCO_3_, 10 g of Difco PDA, 10 g of agar in 1,000 ml of water) as described by Liu et al. (2004b). The cultures were grown for 12–14 days at 21 °C with constant UVB light (Zilla Slimeline desert fixture 50UVB T8 Fluorescent bulb). After pycnidia sporulation, plates with fungal spores were washed with sterile distilled water and scraped with a sterile glass slide. The spore concentration was measured with a hemocytometer and adjusted to a final concentration of 1 × 10^6^ spores ml^−1^ using distilled water. Two drops of Tween 20 (polyoxyethylene sorbitan monolaurate) were added per 100 ml of inoculum to reduce the surface tension.

**Table 1:** Characterized necrotrophic effectors in five isolates of *Parastagonospora nodorum* and percentages of resistant and susceptible hard winter wheat genotypes to these isolates

Isolate ID	Present effectors	Absent effectors	Number of resistant genotypes	Number of intermediate genotypes	Number of susceptible genotypes
OKG16Sn-1	SnToxA, SnTox1, SnTox267, and SnTox5	SnTox3	126 (21%)	195 (32%)	286 (47%)
OKG16Sn-2	SnToxA, SnTox1, SnTox267, SnTox3, and SnTox5		48 (8%)	153 (25%)	407 (67%)
OKG16Sn-9	SnToxA, SnTox3, and SnTox5	SnTox1 and SnTox267	55 (9%)	308 (51%)	242 (40%)
OKG16Sn-13	SnToxA, SnTox1, SnTox267, and SnTox5	SnTox3	53 (9%)	298 (49%)	255 (42%)
OKG16Sn-16	SnToxA, SnTox1, SnTox267, SnTox3, and SnTox5		30 (5%)	315 (52%)	262 (43%)

The 619 wheat genotypes were grown in the greenhouse at 20 °C/18 °C (day/night) with a 16-h photoperiod. The genotypes were planted in 72-cell trays filled with a commercial ‘Ready-Earth’ soil mix (Sun Gro). The susceptible check ‘Gallagher’ was planted as a border in each tray to reduce border effect. For each isolate, the genotypes were tested in a randomized complete block design with 3–4 replicates and 4–5 plants/genotype per replicate (Supplementary Table S1). The differential lines TAM 110, Alsen, LP29, Jerry, Massey, ITMI38, F/G9519, Scoop1, Chinese Spring, BG220, and BG261 were included as checks alongside each experiment (Supplementary Table S3). Jack’s classic all-purpose (20-20-20) fertilizer was applied once a week to the seedlings according to the manufacturer’s instructions. At the second leaf stage, which is approximately 12–13 days post-planting, seedlings were uniformly inoculated using a hand-pressured sprayer until runoff, air-dried for 30 min, then placed in a mist chamber in the dark at 21 °C with 100% relative humidity for 24 h. Thereafter, the plants were transferred to a growth chamber with a 12-h photoperiod, constant temperature of 21 °C, and light intensity of 2,000 umol until disease evaluation. At seven days of post-inoculation, the plants were evaluated for their SNB reactions using a scale of 0-to-5 (Liu et al. 2004b), where 0 = absence of visible lesions (highly resistant); 1 = few penetration points, with lesions exhibiting flecking or small dark spots (resistant); 2 = lesions exhibiting dark spots with little surrounding necrosis or chlorosis (moderately resistant); 3 = dark lesions completely surrounded by necrosis or chlorosis and lesions of 2 to 3 mm (moderately susceptible); 4 = larger necrotic or chlorotic lesions of 4 mm or larger and with little coalescence (susceptible); and 5 = large coalescent lesions with very little remaining green leaf tissue (highly susceptible) (Liu et al. 2004b).

### Effector infiltration

We used five NEs, SnToxA, SnTox1, SnTox3, SnTox5, and SnTox267, produced by *P. nodorum.* SnToxA, SnTox1, and SnTox3 were expressed in *Pichia pastoris* as described in Friesen and Faris (2012). SnTox267 was expressed using a bacterial cell lysate method (Outram et al. 2021), whereas SnTox5 was isolated from a 3-week-old fungal culture filtrate from an avirulent *P. nodorum* strain transformed with *SnTox5* gene (Liu and Friesen 2012; Kariyawasam et al. 2024). The effector infiltration assays were performed at the USDA-ARS Cereal Crops Improvement Research Unit, Fargo, ND, USA. Infiltration was done following the protocol by Friesen and Faris (2012). Plants to be infiltrated were placed under fluorescent lights for 20 min to promote stomata opening. For each NE, two replicates per genotype were planted with two plants per replicate. If there was a discrepancy in the genotype reactions between the two replicates, a third replicate was performed. The lines BG261, Chinese Spring, BG220, BG223, and LP29 were also planted alongside the toxin infiltration experiments as sensitive checks for SnToxA, SnTox1, SnTox3, SnTox267, and SnTox5, respectively (Supplementary Table S1). At the second leaf stage, approximately 20 μl of NE was infiltrated into the second leaf (one leaf per plant) using a needleless 1-ml syringe. The NE was infiltrated into the leaf by pressing the open end of the syringe into the leaf. The liquid was pushed intercellularly via the stomatal openings, leaving a water-soaked appearance on the leaves, marked with a permanent marker. After infiltration, the plants were placed in a growth chamber at 21 °C and 12-h photoperiod. The reactions to NE effectors were scored at 3 days post-infiltration using a 0-to-3 scale, where 0 = no reaction (insensitive), 1 =slight but visible chlorosis, 2 = visible chlorosis/necrosis without tissue collapse, and 3 (highly sensitive) = necrosis with complete tissue collapse (Friesen and Faris 2012). For each NE assay, the susceptible check reaction was considered as a baseline to classify the genotypes into sensitive or insensitive.

Homogeneity of variances among replicates for each NE and *P. nodorum* isolate was assessed using Bartlett’s test in R (Aslam 2020). When Bartlett’s test indicated a violation of variance homogeneity, pairwise comparisons of replicates were performed using the Games-Howell test (Sauder and DeMars 2019). Only replicates that exhibited homogeneous variances were retained and the mean reactions for each genotype were used for subsequent analyses.

### Population structure and linkage disequilibrium

Population structure and linkage disequilibrium (LD) in the wheat panel were performed as described by Lakkakula et al. (2025). Population structure was assessed using principal component analysis (PCA) and based on 34,357 GBS-SNP markers. PCA was performed with the ‘prcomp’ function in R, and the population structure was visualized using the first two principal components (PCs). Linkage disequilibrium (LD) between SNP marker pairs was calculated in TASSEL version 5.2 (Bradbury et al. 2007) as the square of the correlation coefficient (*r*^*2*^). The function ‘geom_smooth’ in the R package ‘ggplot2’ was used to create a locally weighted estimated scatter plot smoother (LOESS) curve (Cleveland 1979) on the LD decay plot.

### Genome-wide association study

Genome-wide association study (GWAS) was performed using phenotypic data, 34,357 filtered and imputed GBS-SNPs, and diagnostic KASP markers for the sensitivity genes *Tsn1-B1*, *Snn1-B1*, *Snn3-B1*, and *Snn3-B2*. GWAS was conducted using three models: the Bayesian information and linkage disequilibrium iteratively nested keyway (BLINK) (Huang et al. 2019), the fixed and random model circulating probability unification (FarmCPU) (Liu et al. 2016), and the mixed linear model (MLM) (Yu et al. 2006) implemented in the Genomic Association and Prediction Integrated Tool v3 (GAPIT 3) (Wang and Zhang 2021) in the R software. The mixed linear model (MLM) controls for population structure and kinship but evaluates one marker at a time, which may reduce its power to detect loci (Zhang et al. 2005; VanRaden 2008; Wen et al. 2018). Multi-locus models such as FarmCPU and BLINK iteratively fit multiple associated markers, improving statistical power while reducing false positive associations (Liu et al. 2016; Huang et al. 2019). It was reported that BLINK outperforms MLM and FarmCPU in detecting true marker trait associations with greater computational efficiency and statistical power (Huang et al. 2019; Wang and Zhang 2021; Sharma et al. 2025).

The GWAS models used in this study incorporated the K matrix and the Q matrix based on PCA. The optimal number of principal components (PCs) included in the Q matrix was based on quantile–quantile (Q–Q) plots that illustrate the deviations between marker observed and expected − log10 (*P*) values (Aoun et al. 2021, 2022; Sharma et al. 2025). The number of PCs tested in the Q matrix was limited to the first four PCs. A threshold of false discovery rate (FDR) (Benjamini and Hochberg 1995) ≤ 0.05 was used to identify significant associations. The R package ‘CMplot’ (https://github.com/YinLiLin/R-CMplot) and the ‘geom_point’ function in the R package ‘ggplot2’ (Wickham and Sievert 2009) were used to create the GWAS Manhattan plots.

### Development of KASP markers associated with response to septoria nodorum blotch

Major loci identified through GWAS that were linked to either unknown resistance/sensitivity genes or to sensitivity genes lacking reliable diagnostic markers in the literature were selected for KASP marker development, to facilitate their potential application in breeding programs. Fifty susceptible genotypes, carrying the susceptible allele of the marker, and fifty resistant genotypes, carrying the resistant allele of the marker, were selected from the GWAS panel to test each of the developed KASP markers in this study. The DNA of the samples was diluted to a concentration of 30 ng µL^−1^. In hexaploid wheat, homoeologous and paralogous regions can have substantial sequence similarity. Thus, locus-specific common primers were designed using the anchoring approach suggested by LGC Biosearch Technologies (https://biosearchassets.blob.core.windows.net/assetsv6/guide_kasp-assay-design-anchoring.pdf). A 200-bp sequence of the SNP of interest in the flanking region was obtained from the IWGSC RefSeq v2.1 (Zhu et al. 2021). This sequence was blasted to identify an anchor point (unique base) at the 3′ end to ensure locus-specific primers. HEX tail 5′-GAAGGTCGGAGTCAACGGATT-3′ and FAM tail 5′-GAAGGTGACCAAGTTCATGCT3′ were added to 5′ end of the SNP marker allele-specific primers. The designed primers were synthesized by Integrated DNA Technologies (Coralville, IA). The KASP reaction was performed using the modified LGC Biosearch Technologies protocol described by Makhoul et al. (2020). PCR and conditions were as described above in the method section ‘Plant materials and genotyping.’

## Results

## Wheat responses against *P. nodorum* isolates at the seedling stage

**Fig. 1:**
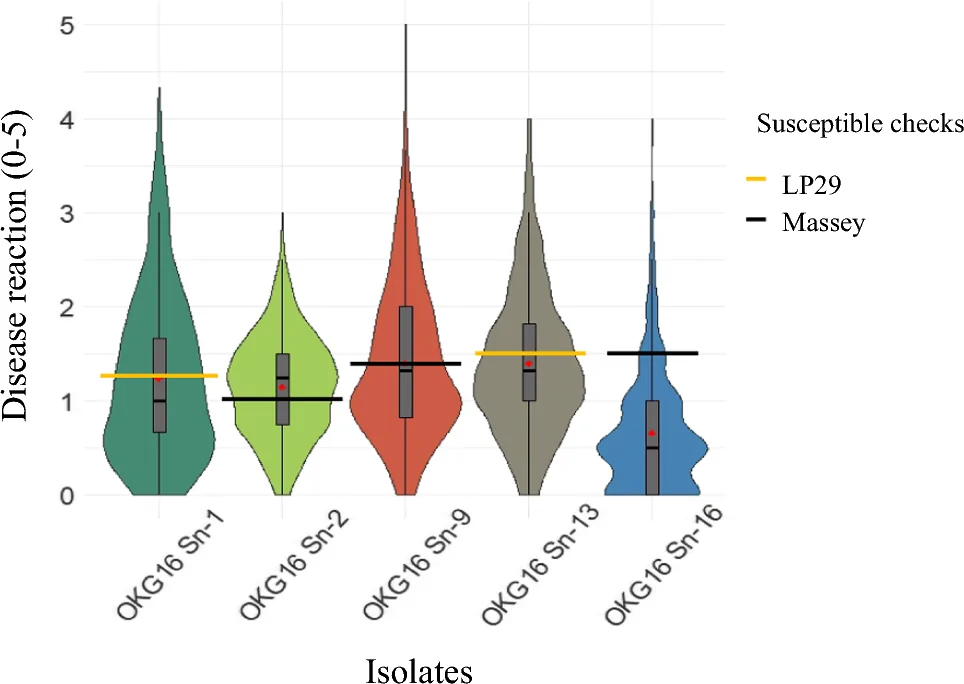
Distribution of disease reactions of 619 hard winter wheat genotypes against five *P. nodorum* isolates at the seedling stage. The red diamonds represent the mean disease reactions in this germplasm. The orange horizontal line indicates the reaction means of the susceptible check ‘LP29’ against isolates OKG16Sn-1 and OKG16Sn-13. The black horizontal line indicates the reaction means of the susceptible check ‘Massey’ against isolates OKG16Sn-2, OKG16Sn-9, and OKG16Sn-16

The reactions of 619 HWW genotypes to inoculation of the *P. nodorum* isolates ranged from 0 (highly resistant) to 5 (highly susceptible) (Fig. 1). The percentages of susceptible genotypes (having a disease reaction equal or higher than the susceptible checks) to isolates OKG16Sn-1, OKG16Sn-2, OKG16-Sn9, OKG16Sn-13, and OKG16Sn-16 were 47%, 67%, 40%, 42%, and 43%, respectively (Table 1). Genotypes with intermediate (moderate) reactions to the five isolates ranged from 25% (against isolate OKG16Sn-2) to 52% (against isolate OKG16Sn-16). There were low percentages of resistant genotypes to *P. nodorum* isolates, ranging from 5 to 21% (Table 1). A total of 75 genotypes had resistant to intermediate reactions to all five isolates (Supplementary Table S4), including the OSU cultivars ‘Uncharted,’ ‘Bentley,’ ‘Big Country,’ and ‘OK Corral.’

### Reactions against *P. nodorum* effectors

**Fig. 2:**
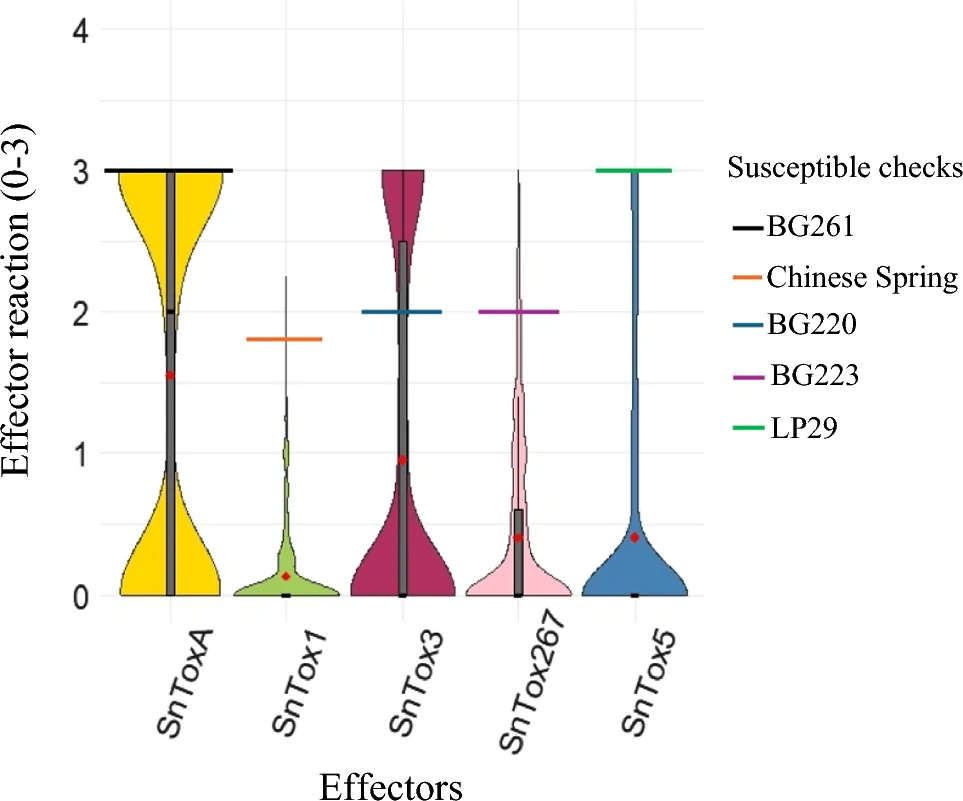
Distribution of reactions of 619 hard winter wheat to five *P. nodorum* effectors at the seedling stage. The red diamonds represent the mean reactions to effectors in this germplasm. The black, orange, blue, purple, and green horizontal lines indicate reaction means of the susceptible checks BG261, Chinese Spring, BG220, BG223, and LP29 to effectors SnToxA, SnTox1, SnTox3, SnTox267, and SnTox5, respectively

Effector infiltration assays against five *P. nodorum* NEs showed that 328 (54%), 12 (2%), 224 (37%), 82 (13%), and 94 (15%) of the genotypes were sensitive to SnToxA, SnTox1, SnTox3, SnTox267, and SnTox5, respectively. Sensitive genotypes had reactions equal or higher than the susceptible checks. The reaction means of the susceptible checks BG261 to SnToxA, Chinese Spring to SnTox1, BG220 to SnTox3, BG223 to SnTox267, and LP29 to SnTox5 were 3, 1.75, 2, 2, and 3, respectively (Fig. 2). These results indicated that the sensitivity genes *Tsn1-B1* and *Snn3-B1/B2* are the most frequent in this germplasm, whereas *Snn1-B1* is the least frequent. In this HWW panel, we identified 153 insensitive genotypes to all five NEs (Supplementary Table S5), of which 37 were also resistant to the five tested *P. nodorum* isolates (Supplementary Table S6). This indicates the presence of additional effectors in *P. nodorum* isolates that are conditioning susceptibility in HWW. The OSU cultivars Big Country, Uncharted, and Bentley were found to be resistant against the five isolates and insensitive to all five tested NEs (Table 2, Supplementary Table S6). Although the cultivar ‘Green Hammer’ was insensitive to the five NEs, it was found to be susceptible to isolates OKG16Sn-1 and OKG16Sn-2 (Table 2), suggesting that these two isolates should carry other unknown effectors, conditioning susceptibility in Green Hammer.

**Table 2:**
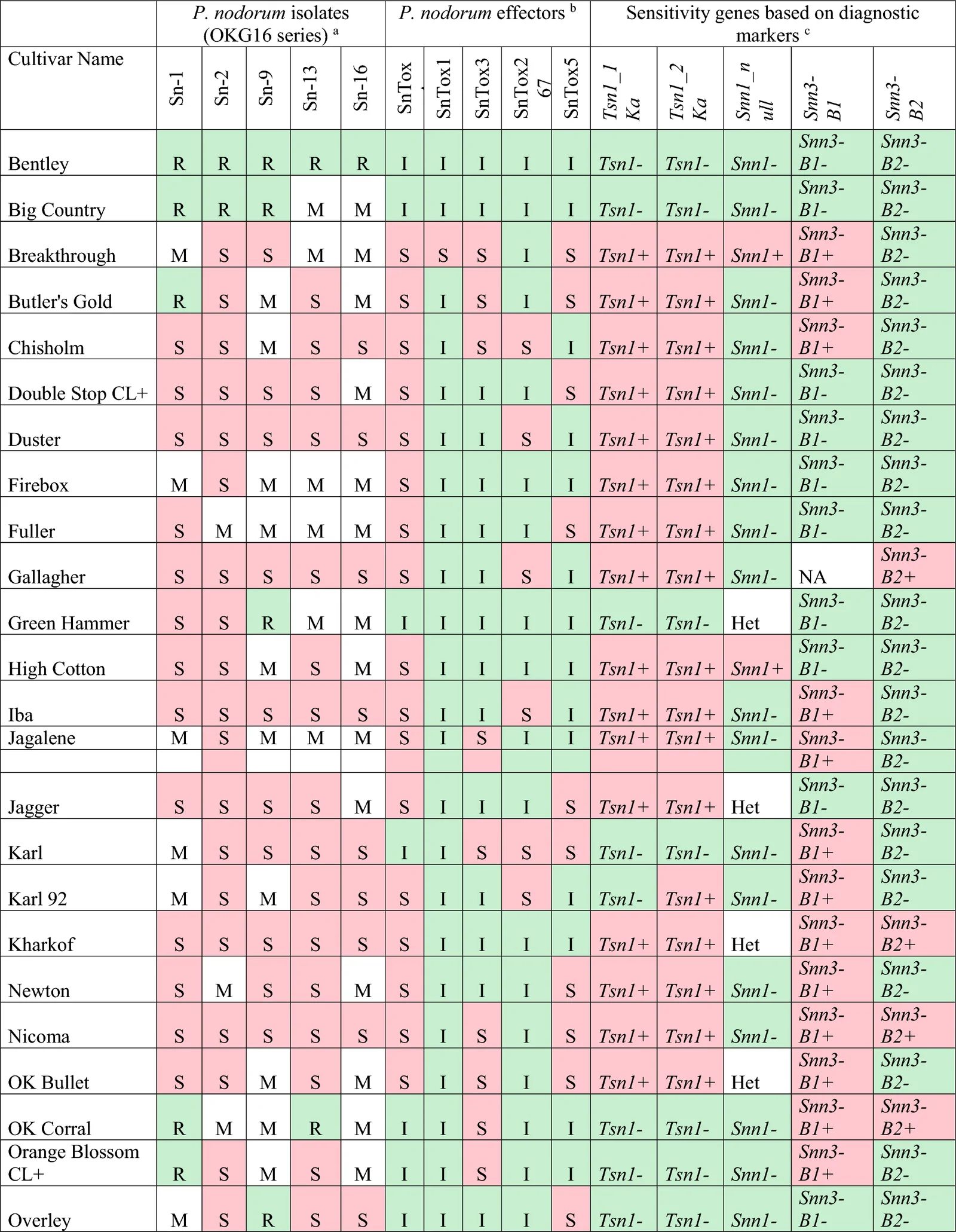

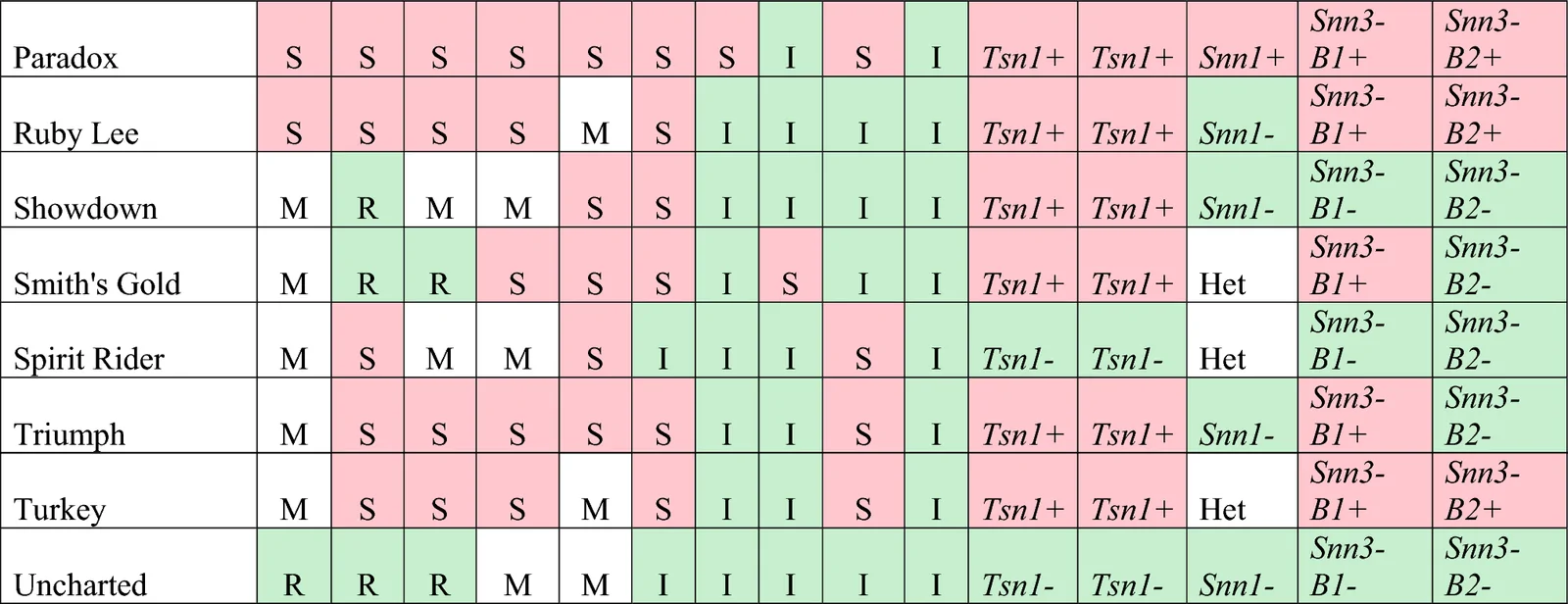
Reactions of 32 hard winter wheat cultivars to *P. nodorum* isolates and effectors with associated sensitivity genes based on molecular markers

^a^ Five *Parastagonospora nodorum* isolates (OKG16 series Sn-1, Sn-2, Sn-9, Sn-13, and Sn-16) were used for seedling evaluation. S = susceptible, M = moderate (or intermediate reactions), R = resistant, NA = data not available due to germination issue

^b^ Purified necrotrophic effectors (SnToxA, SnTox1, SnTox3, SnTox267 and SnTox5) were infiltrated into wheat leaves to determine effector sensitivity. S = sensitive, I = insensitive

^c^ Sensitivity genes were determined using diagnostic markers for *Tsn1-B1*, *Snn1-B1*, and *Snn3-B1/B2* makers. The sensitivity gene is present (+), the sensitivity gene is absent (−), NA: data not available, Het = heterozygous call

### Frequencies of the sensitivity genes *Tsn1-B1*, *Snn1*, *Snn3-B1*, *Snn3-B2* based on KASP markers

Genotyping data for KASP markers (*Tsn1-B1_1Ka* and *Tsn1-B1_2Ka*) associated with *Tsn1-B1* showed that 328 genotypes (54%) carry the *Tsn1-B1* gene, and 280 genotypes (46%) lack this gene. Comparing *Tsn1-B1* marker results with SnToxA infiltration assay data showed that a few genotypes carried *Tsn1-B1* based on markers (n = 5 genotypes for marker *Tsn1-B1_1Ka* and n = 7 genotypes for marker *Tsn1_B1_2Ka*) but were insensitive to SnToxA, suggesting 2% and 3% of false positives for markers *Tsn1-B1_1Ka* and *Tsn1_B1_2Ka*, respectively. Markers revealed that 22% of the genotypes carried *Snn1*-B1, whereas 44% and 24% of the genotypes carried *Snn3-B1* and *Snn3-B2*, respectively. False positive rates for *Snn1-B1* and *Snn3-B1/Snn3-B2* markers were 25% (n = 149 genotypes) and 12% (n = 44 genotypes), respectively. Very low false negatives (genotypes did not carry the sensitivity genes based on markers but sensitive to the corresponding NEs) were identified with these markers; 2% (n = 5 genotypes) and 1% (n = 4 genotypes) for *Tsn1-B1* markers *Tsn1-B1_1Ka* and *Tsn1-B1_2Ka*, respectively, 0% for *Snn1-B1* marker, and 3% (n=6 genotypes) for *Snn3-B1/Snn3-B2* markers. The overall accuracy of markers *Tsn1-B1_1Ka*, *Tsn1-B1_2Ka*, *Snn1-B1*, and *Snn3-B1/Snn3-B2* was 98%, 98%, 75%, and 92%, respectively.

### Linkage disequilibrium and PCA

Of the filtered 34,357 SNPs, 11,878 (34.6%) were mapped to the A genome, 16,150 (47%) to the B genome, 5,884 (17.1%) to the D genome, and 445 (1.3%) were unaligned (UN) to a chromosome. SNP numbers ranged from 322 on chromosome 4D to 3,685 on chromosome 2D. The highest density of SNP markers in the 1 Mb window was observed on the A genome, whereas the lowest density of SNP markers was observed on the D genome (Supplementary Fig. S2). The genome-wise LD dropped to an *r*^2^ of 0.25 within 2.0 Mb on average (Supplementary Fig. S3). LD decayed to 0.25 at ∼1.2 Mb on average for genome A, at 2.5 Mb on average for genome B, and at 6.8 Mb on average for genome D (Supplementary Fig. S4). PCA using 34,357 SNPs showed low to moderate structure in the 619 HWW genotypes, where the first two PCs, PC1 and PC2, explained 10.7% and 6.0% of the genetic variation, respectively (Supplementary Fig. S5). The first 10 PCs accounted cumulatively for 35.5% of the variation.

### GWAS model selection

The most suitable GWAS model for each trait was selected based on the examination of the Q–Q plots. For all traits, the K matrix was included in the GWAS models, and the optimal number of PCs in the Q matrix varied by trait (Supplementary Figs. S6 and S7). In the MLM model, the Q matrix was excluded (no PCs) for all traits except responses to isolates OKG16Sn-2 and OKG16Sn-9, where the first four PCs were included. For FarmCPU model, the first two PCs were included in the Q matrix for responses to all isolates except OKG16Sn-16, for which no PCs were included. Similarly, no PCs were included for responses to all NEs except SnTox267, for which the first two PCs were incorporated in the Q matrix of the model. In the BLINK model, no PCs were included for responses to isolates OKG16Sn-1, OKG16Sn-9, OKG16Sn-13, and OKG16Sn-16, as well as to the NEs SnToxA, SnTox1, and SnTox5, while the Q matrix included the first two PCs for responses to the NEs SnTox3 and SnTox267, and the first four PCs for response to isolate OKG16Sn-2.

### Markers associated with responses to *P. nodorum* isolates and necrotrophic effectors

**Fig. 3:**
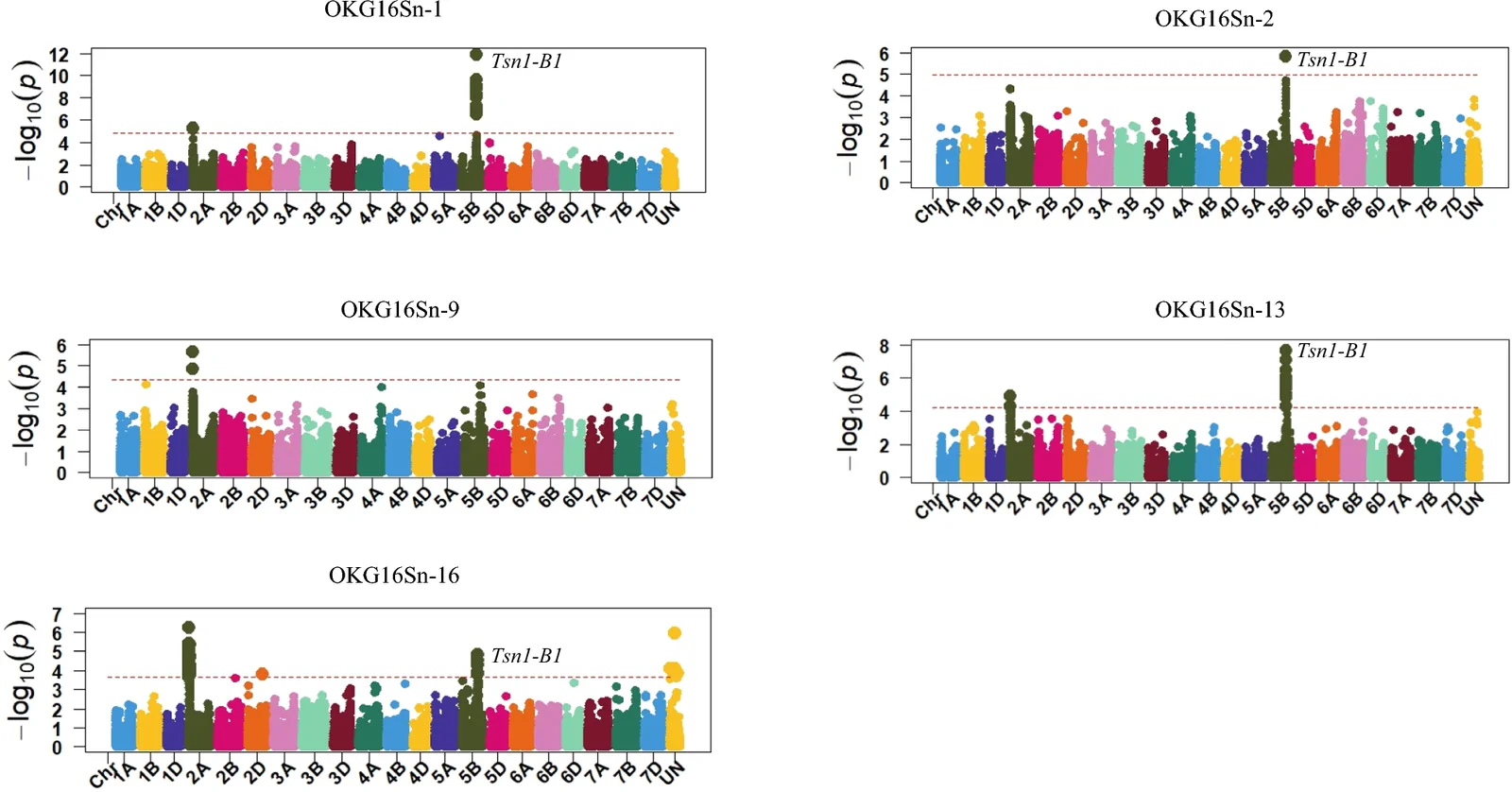
Manhattan plots showing significant markers associated with responses to five *P. nodorum* isolates using the MLM model. The horizontal red line indicates significance levels at a false discovery rate ≤ 0.05

**Fig. 4:**
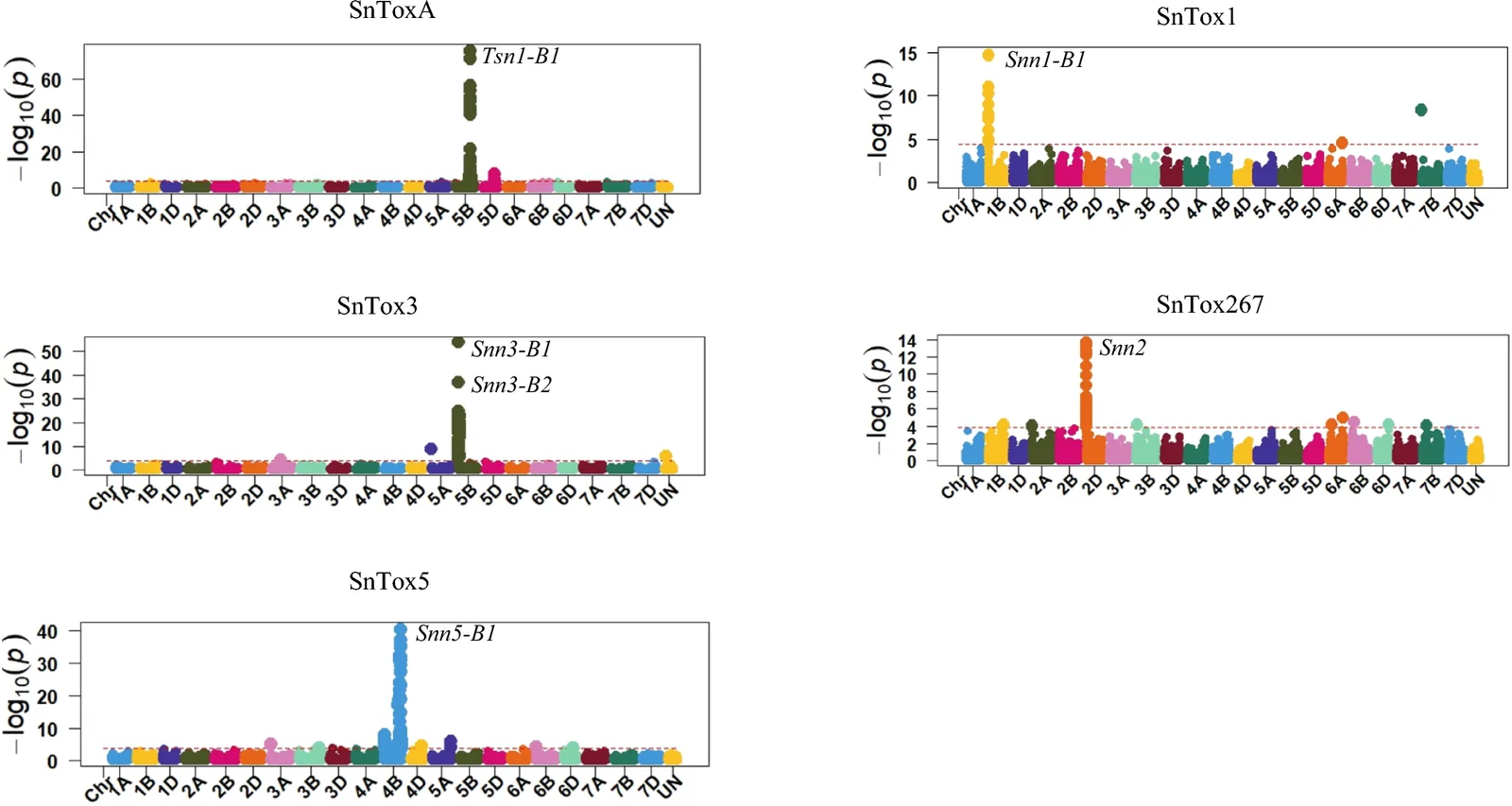
Manhattan plots showing significant markers associated with responses to five *P. nodorum* effectors using the MLM model. The horizontal red line indicates significance levels at a false discovery rate ≤ 0.05

Significant markers based on the GWAS single-locus model MLM are presented in Figs. 3 and 4 and Supplementary Table S7. Significant markers based on the GWAS FarmCPU model are presented in Supplementary Table S8, whereas significant markers based on the GWAS BLINK model are presented in Table 3, Supplementary Figs. S8 and S9. Based on the BLINK model, there were 23 significant loci associated with responses to the *P. nodorum* isolates, and 49 loci associated with responses to the NEs (Table 3). The highest number of significant SNPs associated with responses to *P. nodorum* isolates was positioned on chromosome arm 5BL, which corresponds to the position of *Tsn1-B1* gene (Table 3, Fig. 3, Supplementary Tables S7 and S8, Supplementary Fig. S8). This indicates the importance of this gene in explaining susceptibility to *P. nodorum* isolates, which all carry the NE SnToxA. *Snn2* linked to the marker *S2D_17032294* on chromosome 2DS was also found to be associated with SNB susceptibility to isolates OKG16Sn-2 and OKG16Sn-13.

**Table 3:** Summary of significant markers associated with septoria nodorum blotch response at the seedling stage using the BLINK model

Trait ^a^	Marker ^b^	Alleles ^c^	*P* value	MAF ^d^	FDR ^e^	Effect ^f^	Markers identified using other GWAS models	Possible *Sn* gene
OKG16Sn-1	*S2A_6917223*	**C**/T	5.26E−15	0.26	9.04E−11	0.27	MLM	
OKG16Sn-1	***Tsn1-B1_1Ka***	C/**T**	5.97E−27	0.47	2.05E−22	− 0.36	MLM + FarmCPU	*Tsn1-B1*
OKG16Sn-2	*S2A_18073519*	G/**T**	4E−08	0.48	1.37E−03	− 0.13		
OKG16Sn-2	***S2D_17032294***	**C**/T	2.47E−06	0.13	2.12E−02	0.14		*Snn2*
OKG16Sn-2	***Tsn1-B1_1Ka***	C/**T**	5.24E−07	0.47	6.00E−03	− 0.10	MLM	*Tsn1-B1*
OKG16Sn-2	*S6D_9896779*	**G**/A	1.2E−07	0.06	2.07E−03	0.22		
OKG16Sn-9	*S2A_20229145*	A/**G**	5.77E−13	0.45	1.98E−08	− 0.24	MLM + FarmCPU	
OKG16Sn-9	*S5B_536277618*	**T**/A	3.36E−06	0.06	2.89E−02	0.38		
OKG16Sn-9	*S6B_651138667*	**A**/G	3.57E−08	0.05	4.09E−04	0.42		
OKG16Sn-9	*S7B_730920233*	**T**/A	2.05E−11	0.30	3.52E−07	0.23		
OKG16Sn-13	*S1A_47826221*	T/**C**	2.28E−09	0.40	1.95E−05	− 0.15		
OKG16Sn-13	*S2A_16181637*	A/**T**	5.56E−22	0.50	1.91E−17	− 0.24	MLM	
OKG16Sn-13	*S2B_24543377*	**T**/C	1.5E−07	0.46	6.45E−04	0.12	FarmCPU	
OKG16Sn-13	***S2D_17032294***	**C**/T	1.16E−07	0.14	5.71E−04	0.21	FarmCPU	*Snn2*
OKG16Sn-13	*S3B_508585032*	**G**/A	5.52E−13	0.49	6.33E−09	0.18	FarmCPU	
OKG16Sn-13	*Tsn1-B1_2Ka*	C/**T**	2.79E−18	0.46	4.79E−14	− 0.22		*Tsn1-B1*
OKG16Sn-13	*S5B_598897426*	**T**/A	6.07E−08	0.15	3.48E−04	0.17		
OKG16Sn-13	*S5B_693810821*	**G**/A	2.11E−06	0.18	7.25E−03	0.16		
OKG16Sn-13	*S6B_7978415*	**G**/A	1.61E−06	0.19	6.13E−03	0.14	FarmCPU	
OKG16Sn-13	*S7B_712245127*	**A**/G	1.35E−05	0.40	4.22E−02	0.11		
OKG16Sn-13	*SUN_47146069*	T/**C**	1.37E−08	0.08	9.41E−05	− 0.25		
OKG16Sn-16	*S2A_2543865*	G/**A**	5.86E−21	0.46	2.02E−16	− 0.26	MLM + FarmCPU	
OKG16Sn-16	*S2A_9619593*	**A**/G	1.33E−10	0.40	1.52E−06	0.18	MLM + FarmCPU	
OKG16Sn-16	*S5A_11560883*	G/**A**	1.93E−06	0.44	1.33E−02	− 0.11	FarmCPU	
OKG16Sn-16	***Tsn1-B1_1Ka***	C/**T**	1.31E−12	0.47	2.24E−08	− 0.19		*Tsn1-B1*
OKG16Sn-16	*S7B_727439174*	**G**/A	1.19E−07	0.15	1.03E−03	0.18		
SnToxA	***Tsn1-B1_1Ka***	C/**T**	6.33E−228	0.47	2.18E−223	− 1.29	MLM + FarmCPU	*Tsn1-B1*
SnToxA	*S5B_549870920*	T/**G**	3.81E−10	0.45	6.55E−06	− 0.29	MLM	*Tsn1-B1*
SnToxA	*S5B_550553444*	C/**T**	1.42E−06	0.42	1.22E−02	− 0.18	MLM	*Tsn1-B1*
SnToxA	*S5B_565520768*	G/**T**	4.83E−07	0.39	5.53E−03	− 0.10	MLM	*Tsn1-B1*
SnTox1	*S1A_29327746*	**G**/A	9.19E−08	0.21	1.05E−03	0.06		
SnTox1	*Snn1-B1*	**C**/T	1.09E−26	0.26	3.74E−22	0.13	MLM + FarmCPU	*Snn1-B1*
SnTox1	*S2B_808851471*	**A**/G	5.92E−07	0.08	4.07E−03	0.09		
SnTox1	*S6A_173098821*	**C**/T	4.44E−07	0.05	3.81E−03	0.11	FarmCPU	
SnTox1	*S7B_27224473*	**C**/G	9.6E−13	0.07	1.65E−08	0.16	MLM + FarmCPU	
SnTox3	*S3B_52279581*	A/**G**	5.9E−10	0.16	4.05E−06	− 0.23		
SnTox3	*S5B_961630*	**A**/C	5.07E−21	0.37	5.81E−17	0.25	MLM + FarmCPU	
SnTox3	*S5B_6473894*	A/**G**	7.73E−32	0.29	1.33E−27	−0.39	MLM + FarmCPU	
SnTox3	*S5B_6477908*	C/**T**	2.74E−09	0.23	1.57E−05	− 0.24	MLM	
SnTox3	*S5B_6883447*	T/**G**	1.79E−06	0.37	7.69E−03	− 0.15	MLM	
SnTox3	*Snn3-B1*	**C**/T	1.25E−73	0.43	4.28E−69	0.61	MLM + FarmCPU	*Snn3-B1*
SnTox3	*S5B_14688078*	**T**/C	5.6E−14	0.27	4.81E−10	0.22	MLM + FarmCPU	
SnTox3	*S7D_553955259*	G/**C**	1.08E−07	0.10	5.31E−04	− 0.22		
SnTox267	*S1A_461319899*	G/**A**	1.61E−07	0.19	8.31E−04	− 0.12		
SnTox267	*S1B_536259624*	G/**A**	1.36E−05	0.18	3.11E−02	− 0.09		
SnTox267	*S2A_624084*	**T**/C	8.52E−10	0.11	9.76E−06	0.19	MLM + FarmCPU	
SnTox267	*S2A_18513359*	**A**/G	3.03E−07	0.48	1.16E−03	0.09	FarmCPU	
SnTox267	*S2A_784930308*	**T**/A	3.02E−07	0.36	1.16E−03	0.08		
SnTox267	*S2D_11282990*	**C**/G	9.18E−17	0.43	3.16E−12	0.21	MLM	
SnTox267	*S2D_16294137*	**G**/A	2.99E−08	0.27	2.57E−04	0.14	MLM	*Snn2*
SnTox267	*S4A_741637942*	**C**/A	4.84E−07	0.06	1.51E−03	0.14	FarmCPU	
SnTox267	*S5A_584897357*	C/**T**	9.59E−07	0.08	2.75E−03	− 0.15	FarmCPU	
SnTox267	*S6A_573111444*	**C**/G	4.61E−08	0.11	3.17E−04	0.17	MLM + FarmCPU	
SnTox267	*S6A_622110677*	A/**G**	1.69E−07	0.43	8.31E−04	− 0.07		
SnTox267	*S6B_49975604*	**C**/T	3.16E−06	0.20	7.75E−03	0.12	FarmCPU	
SnTox267	*S6B_121323589*	**C**/T	2.8E−06	0.05	7.41E−03	0.17	MLM + FarmCPU	
SnTox267	*S7B_177204116*	T/**C**	7.96E−10	0.33	9.76E−06	− 0.11	MLM + FarmCPU	
SnTox267	*S7D_112398508*	**C**/T	4.77E−07	0.08	1.51E−03	− 0.17		
SnTox5	*S1B_484765780*	**A**/G	2.35E−06	0.16	5.04E−03	0.12		
SnTox5	*S1B_676515304*	**C**/T	9.24E−08	0.08	2.44E−04	0.18		
SnTox5	*S1D_3575130*	T/**A**	4.00E−10	0.06	1.67E−06	− 0.26	FarmCPU	
SnTox5	*S2A_142723679*	**C**/A	1.68E−06	0.10	3.85E−03	0.12		
SnTox5	*S3A_13031539*	**A**/G	1.36E−14	0.06	1.56E−10	0.37	MLM	
SnTox5	*S3A_624107610*	C/**T**	4.38E−10	0.13	1.67E−06	− 0.18		
SnTox5	*S3A_651783353*	**C**/T	8.42E−07	0.11	2.07E−03	0.14		
SnTox5	*S3B_21344739*	A/**T**	9.43E−09	0.11	2.95E−05	− 0.16		
SnTox5	*S3B_145494706*	T/**C**	4.30E−11	0.16	2.46E−07	− 0.17		
SnTox5	*S4B_543724873*	C/**T**	4.08E−13	0.10	3.50E−09	− 0.21	MLM	
SnTox5	*S4B_628548633*	**C**/A	2.66E−28	0.11	4.56E−24	0.46	MLM + FarmCPU	*Snn5-B1*
SnTox5	*S4B_636310716*	**T**/C	1.43E−60	0.27	4.91E−56	0.41	MLM + FarmCPU	
SnTox5	*S4B_643615365*	**C**/G	1.70E−08	0.15	4.87E−05	0.18	MLM + FarmCPU	*Snn5-B1*
SnTox5	*S4B_652333708*	**C**/T	5.10E−12	0.12	3.50E−08	0.17	MLM + FarmCPU	
SnTox5	*S5A_7129378*	C/**T**	5.11E−09	0.11	1.76E−05	− 0.16		
SnTox5	*S5A_694630213*	**G**/A	1.14E−10	0.15	5.57E−07	0.16		
SnTox5	*S6A_462661785*	**G**/T	6.81E−06	0.05	1.38E−02	0.17	FarmCPU	

^a^ Traits as *P. nodorum* isolates and effectors at the seedling stage

^b^ Markers in bold were identified with more than one trait. Marker name has the wheat chromosome, which follows the letter ‘S’ and the physical positions of markers (based on the Wheat Chinese Spring IWGSC RefSeq v2.1; Zhu et al. 2021), which follows the underscore ‘_’

^c^ Marker major/minor allele, the allele in bold is associated with septoria nodorum blotch resistance (favorable allele)

^d^ Minor allele frequency

^e^ False discovery rate

^f^ Negative marker effect: the minor allele is associated with resistance; positive marker effect: the major allele is associated with resistance

Another locus on chromosome arm 2AS and designated here as *Qsnb.osu-2AS* was found to be associated with responses to all isolates. *Qsnb.osu-2AS* effects on responses to isolates OKG16Sn-2, OKG16Sn-13, and OKG16Sn-16 exceed that of the *Tsn1* locus (Table 3). This locus was not reported to be associated with any known SNB sensitivity/resistance genes. The markers in this region were in high LD, as illustrated in Supplementary Fig. S10, indicating that this locus was associated with response to all five *P. nodorum* isolates. Resistant allele frequencies for *Qsnb.osu-2AS* were present in high frequency in this germplasm (~ 45–60%, depending on the significant markers on 2AS, Table 3). Significant markers associated with *Qsnb.osu-2AS* spanned a physical region of 17.69 Mb (marker *S2A_2543865* to *S2A_20229145*). Based on the Wheat Chinese Spring IWGSC RefSeq v2.1, there were 268 high-confidence genes, with multiple genes involved in disease resistance and defense-related pathways (Supplementary Table S9). In addition to the loci on chromosome arms 5BL, 2AS, and 2DS, the BLINK model identified other unknown loci associated with responses to isolates OKG16Sn-2, OKG16Sn-9, OKG16Sn-13, and OKG16Sn-16 located on chromosome arms 1AS, 2BS, 3BL, 5AS, 5AL, 6BS, 6BL, 6DS, and 7BL (Table 3, Supplementary Fig. S8).

GWAS identified the sensitivity genes *Tsn1-B1* (on chromosome 5BL), *Snn1-B1* (on chromosome 1BS), *Snn3-B1/Snn3-B2* (on chromosome 5BS), *Snn2* (on chromosome 2DS), *Snn5-B1* (on chromosome 4BL) to be associated with responses to the NE effectors SnToxA, SnTox1, SnTox3, SnTox267, and SnTox5, respectively (Table 3, Fig. 4). In addition to these genes, the BLINK model identified other unknown loci associated with responses to SnTox1, SnTox3, SnTox267, and SnTox5 and positioned on chromosome arms 1AS, 1AL, 1BL, 1DS, 2AL, 2BL, 3AS, 3AL, 3BS, 3BL, 4AL, 4BL, 5AS, 5AL, 6AL, 6BS, 6BL, 7BS, 7BL, and 7DL (Table 3, Supplementary Fig. S9).

### Development and validation of KASP markers associated with response to septoria nodorum blotch

**Fig. 5:**
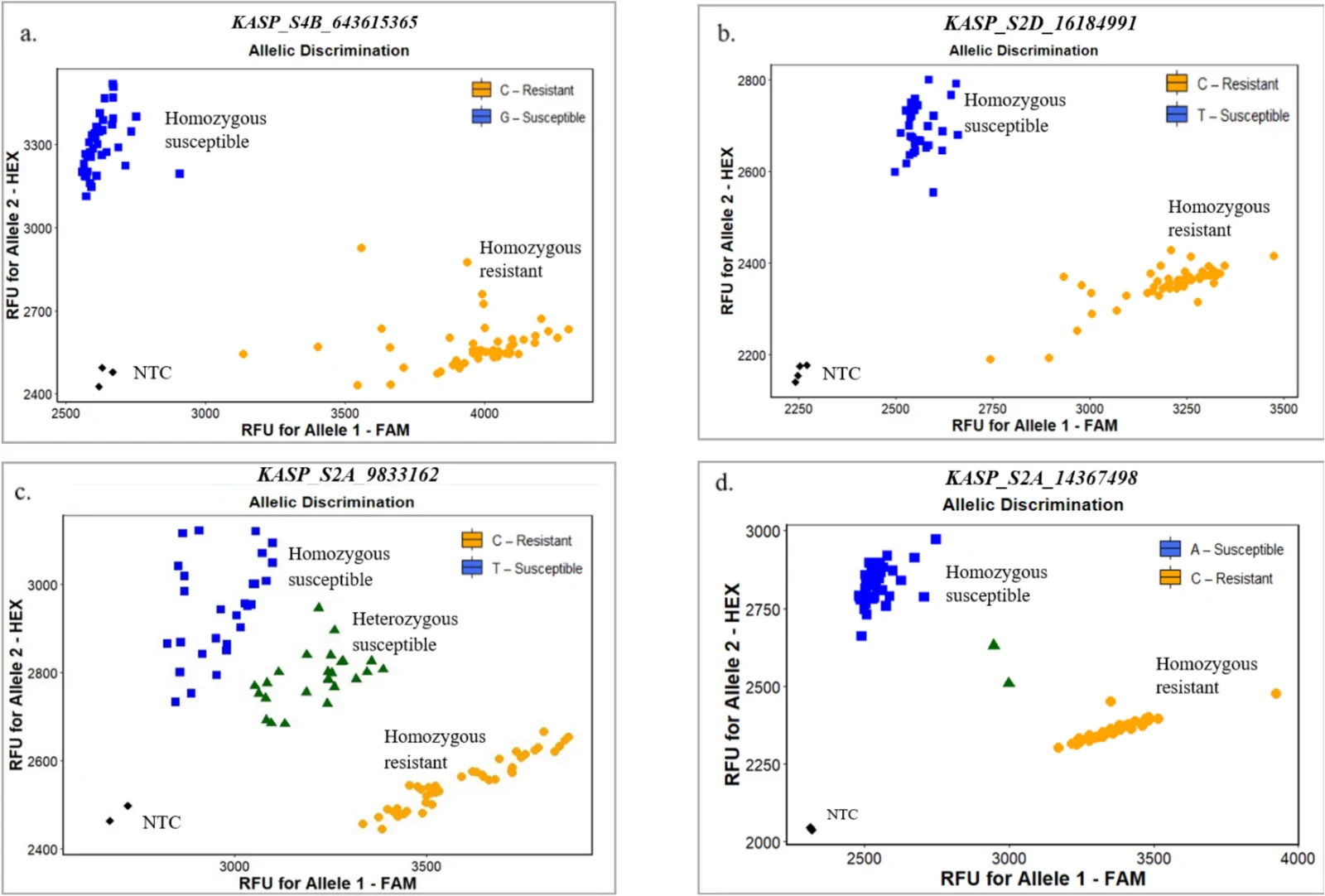
Genotyping scatter plots for the kompetitive allele-specific PCR (KASP) markers, *KASP_S4B_643615365* (linked to *Snn5-B1*), *KASP_ S2D_16184991* (linked to *Snn2*), and *KASP_S2A_9833162* and *KASP_S2A_14367498* (both linked to *Qsnb.osu-2AS*)

As no diagnostic marker for *Snn5-B1* is currently available, we targeted the region of *Snn5-B1* gene on chromosome arm 4BL by selecting the most significant SNP markers from the three tested GWAS models for KASP development (*S4B_636310716*, *S4B_628548633*, *S4B_652333708*, and *S4B_643615365*). Of these four SNPs, we successfully developed a KASP marker for *S4B_643615365*, here designated as *KASP_ S4B_643615365*. The primer sequences for the *KASP_ S4B_643615365* are provided in Table 4. *KASP_ S4B_643615365* was validated on 100 genotypes from this GWAS panel, with 50 resistant genotypes carrying the allele (C) and 50 susceptible genotypes carrying the allele (G) (Fig. 5a). Genotype calls for the *KASP_ S4B_643615365* agreed with those of the GBS-SNP marker *S4B_643615365* for all the 100 tested genotypes. *KASP_ S4B_643615365* had an accuracy of 89% in predicting sensitivity/insensitivity to SnTox5 based on the entire GWAS panel.

**Table 4:** Kompetitive allele-specific PCR (KASP) marker developed from the most significant genotyping-by-sequencing (GBS) SNPs linked to *Snn5-B1* on chromosome 4BL, *Snn2* on chromosome 2DS, and *Qsnb.osu-2AS* on chromosome arm 2AS

KASP marker	Primer	Sequence (5′−3′)	Amplicon size (bp)
*KASP_S4B_643615365*	Allele-specific reverse primer-1	HEX_GTGTTTGGCTCCTATGCCGA**C**	79
	Allele-specific reverse primer-2	FAM_GTGTTTGGCTCCTATGCCGA**G**	
	Common forward primer	CAAGTTTGCCGGTGTGTGCC	
*KASP_ S2D_16184991*	Allele-specific forward primer-1	FAM_AGTGGCGGGTCGCGTC**C**	109
	Allele-specific forward primer-2	HEX_AGTGGCGGGTCGCGTC**T**	
	Common reverse primer	TCCGACGAAGCAGTTCCATCCAG	
*KASP_S2A_9833162*	Allele-specific reverse primer-1	FAM_TGGAGCCAGGGAGCACCAT**G**	68
	Allele-specific reverse primer-2	HEX_TGGAGCCAGGGAGCACCAT**A**	
	Common forward primer	GCAGACAGGAGATGTATGTACCAC	
*KASP_S2A_14367498*	Allele-specific reverse primer-1	FAM_CTTTAGCATATGCTTTGGCG**G**	49
	Allele-specific reverse primer-2	HEX_CTTTAGCATATGCTTTGGCG**T**	
	Common forward primer	GATCAGCCTCGGTTCATACA	

*KASP_S4B_643615365*, the susceptible allele (G) is labeled with HEX and the resistant allele (C) is labeled with FAM.

*KASP_ S2D_16184991*, the resistant allele (C) is labeled with FAM, and the susceptible allele (T) is labeled with HEX.

*KASP_S2A_9833162*, the susceptible allele (T) is labeled with HEX and the resistant allele (C) is labeled with FAM.

*KASP_S2A_14367498*, the susceptible allele (A) is labeled with HEX and the resistant allele (C) is labeled

Resistant and susceptible alleles of the SNPs are highlighted in bold

As no diagnostic marker for *Snn2* is currently available, we targeted the locus of the *Snn2* gene on chromosome arm 2DS by selecting the most significant SNP markers from the three tested GWAS models for KASP development (*S2D_16294137*, *S2D_16383299*, *S2D_16184991*, and *S2D_16963560*). Of these four SNPs, we successfully developed a KASP marker for *S2D_16184991*, here designated as *KASP_ S2D_16184991*. The primer sequences for the *KASP_ S2D_16184991* are provided in Table 4. *KASP_ S2D_16184991* was validated on 100 genotypes from the OSU GWAS panel, with 50 resistant genotypes carrying the allele (C) and 50 susceptible genotypes carrying the allele (T) (Fig. 5b). Genotype calls for the *KASP_ S2D_16184991* agreed with those of the GBS-SNP marker *S2D_16184991* for all 100 tested genotypes. *KASP_ S2D_16184991* had an accuracy of 80% in predicting sensitivity/insensitivity to SnTox267 based on the entire GWAS panel.

The novel locus on chromosome arm 2AS, designated here as *Qsnb.osu-2AS*, was found to be associated with response to all five *P. nodorum* isolates tested in this study. To develop KASP markers, we targeted the most significant SNPs on chromosome 2AS from the three tested GWAS models for KASP development (*S2A_14367498*, *S2A_2543865*, *S2A_9833162*, and *S2A_17970052*). Of these four SNPs, we successfully developed KASP markers for *S2A_9833162* and *S2A_14367498*, here designated as *KASP_ S2A_9833162* and *KASP_S2A_14367498*, respectively. The primer sequences for the *KASP_ S2A_9833162* and *KASP_S2A_14367498* are provided in Table 4. Both of these markers were validated each on 100 genotypes from the OSU GWAS panel, with 50 resistant genotypes carrying the allele (C) and 50 susceptible genotypes carrying the allele (T) (Fig. 5c, d). Genotype calls for the *KASP_ S2A_9833162* agreed with those of the GBS-SNP marker *S2A_9833162* for all 50 tested resistant genotypes. The susceptible genotypes, which were carrying the susceptible allele (T) according to the GBS-SNP marker, showed a mixture of homozygous susceptible allele and heterozygous calls based on the developed KASP marker (Fig. 5c). Genotype calls for *KASP_ S2A_14367498* agreed with those of the GBS-SNP marker *S2A_14367498*, except two samples that were classified as heterozygous by the KASP assay but as homozygous resistant by the SNP marker (Fig. 5d).

## Discussion

The increased incidence of septoria nodorum blotch in HWW production regions in the Great Plains (Aoun and Carver 2024) has made breeding for SNB resistance a priority. This is the first comprehensive study of SNB susceptibility/resistance in contemporary HWW, integrating multiple approaches: evaluating host reactions to *P. nodorum* isolates and NEs, identifying characterized SNB sensitivity genes using diagnostic markers, and validating known sensitivity genes while uncovering novel resistance/susceptibility loci through genome-wide association studies (GWAS).

Seedling evaluations of 619 HWW breeding lines and cultivars to *P. nodorum* isolates showed that up to 67% of the genotypes were susceptible. Richards et al. (2019) found that *P. nodorum* isolates collected from Oklahoma were clustered separately from all other isolates collected from other US states. Furthermore, all Oklahoma isolates in this survey carried *SnToxA*, which interacts with *Tsn1* to cause susceptibility. In this study, we found that 54% of this HWW panel were sensitive to SnToxA; thus, the frequency of *Tsn1* should be reduced in HWW to enhance SNB resistance. *Tsn1*, which encodes a protein with integrated serine/threonine protein kinase (PK), nucleotide binding, and leucine-rich repeat (NLR) domains (Faris et al. 2010), also causes susceptibility to other fungal diseases, including tan spot (*Pyrenophora tritici-repentis*) and spot blotch (*Bipolaris sorokiniana*), making it the first sensitivity gene to target with marker-assisted elimination. Previous studies showed *Tsn1* is present in 32–76% of spring and winter wheat panels (Waters et al. 2011; Tan et al. 2014; Bertucci et al. 2014; Ruud et al. 2018; Hafez et al. 2020; AlTameemi et al. 2021; Peters Haugrud et al. 2023; Szabo-Hever et al. 2025b). The high frequency of *Tsn1* in different wheat classes is likely due to its genetic linkage with other genes that confer desirable traits in wheat, or this gene may have a function other than conferring sensitivity to SnToxA (Richards et al. 2019; Cowger et al. 2020; Hafez et al. 2020; AlTameemi et al. 2021; Szabo-Hever et al. 2025a). Faris et al. (2010b) demonstrated that the *Tsn1* gene originated from a gene fusion event that might be functioning in the diploid B genome progenitor of polyploid wheat. Consequently, *Tsn1* was perhaps transferred to hexaploid wheat from tetraploid wheat by either amphiploidization or secondary hybridization events.

Sensitivity to SnTox3 was present in 37% of this HWW panel, making *Snn3* another target gene to eliminate in HWW breeding programs. The frequency of SnTox3 sensitivity was lower compared to that (52%) reported in a hard red spring wheat panel and higher compared to that observed in a global durum wheat panel (22%) (Szabo-Hever et al. 2025b). *Tsn1*-*SnToxA* and *Snn3*-*SnTox3* interactions were found to play an important role in SNB sensitivity in other spring and winter wheat panels (Adhikari et al. 2011; Gurung et al. 2014; Liu et al. 2015; Phan et al. 2018; Ruud et al. 2019; Halder et al. 2019; AlTameemi et al. 2021; Peters Haugrud et al. 2023).

The frequency of SnTox1 sensitivity was low (2%) in this HWW panel, which is lower than that observed in panels of hard red spring wheat (28%; Szabo-Hever et al. 2025b), winter wheat (37%; Peters Haugrud et al. 2023), and durum wheat (65%; Szabo-Hever et al. 2025b). The low frequency of SnTox1 could be due to the absence of a functional *Snn1-B1* allele, which was evident based on the false positive rate of 25% of the diagnostic marker available for this allele. *Snn1* could have gone through mutation deletions, and inversions, resulting in the loss of its sensitivity; thus, the available *Snn1-B1* diagnostic marker could not detect these diverse structural variations (Seneviratne et al. 2024a). Shi et al. (2016a, b) showed that a homoeoallele of *Snn1* is present on chromosome 1D of hexaploid wheat, which could compensate for the loss of *Snn1* on 1B. The lack of significant associations on chromosome 1D for response to SnTox1 could be due to the low allele frequency of the D genome copy of *Snn1*.

Sensitivity to SnTox267 and SnTox5 in this HWW panel was 13% and 15%, respectively. These frequencies were lower compared to those reported in a winter wheat panel, which were 39 and 42% sensitivity to SnTox267 and SnTox5, respectively (Peters Haugrud et al. 2023). Szabo-Hever et al. (2025b) reported that sensitivity to SnTox267and SnTox5 in a hard red spring wheat panel was 54 and 12%, respectively. Our GWAS in response to SnTox267 and isolates OKG16Sn-2 and OKG16Sn-13 identified significant associations linked to *Snn2* (on chromosome 2DS; Friesen et al. 2007), but not to *Snn6* (on chromosome 6AL; Gao et al. 2015) and *Snn7* (on chromosome 2DL; Shi et al. 2015). These results agreed with those of Peters Haugrud et al. (2023), where no associations were found for *Snn6* and *Snn7* genes in a global winter wheat collection. In addition to *Snn2*, Szabo-Hever et al. (2025b) identified associations on chromosome 2DL for response to SnTox267 in hard red spring wheat, which may correspond to *Snn7*, but did not show any associations linked to *Snn6*. *Snn7* was identified and mapped in Chinese Spring’ (CS)–Timstein (CS-Tm) disomic chromosome substitution lines (Gao et al. 2015; Shi et al. 2015b). The sensitivity gene *Snn6* is not common in hexaploid wheat and was identified only in the synthetic wheat line W-7984 (Gao et al. 2015).

Our GWAS reported that sensitivity to SnTox5 was mainly attributed to the presence of *Snn5-B1* on chromosome 4BL. The GWAS BLINK model also identified other loci on chromosome 1B, 1D, 2 A, 3 A, 3B, 5 A, and 6 A to be associated with response to SnTox5. Additional loci (other than *Snn5*) on chromosomes 5 A and 5B were also found to be associated with response to SnTox5 in winter wheat (Peters Haugrud et al. 2023). Further work is needed to determine the interaction between SnTox5 and any of these GWAS identified loci.

Although isolate OKG16Sn-9 did not produce the NEs SnTox1 or SnTox267, it produced the highest disease scores on susceptible lines, reaching a severity score of 5 (a level not observed with any of the other isolates). Phan et al. (2016) reported that the *Snn1*−SnTox1 interaction is epistatic with *Snn3*−SnTox3 interaction, and higher expression of SnTox3 in *P. nodorum* was observed in the absence of SnTox1. Peters Haugrud et al. (2019) also reported antagonistic epistasis between SnTox1 and SnToxA, where *SnTox1* gene expression level in isolate Sn2000 was lower when SnToxA was produced. This may explain the lack of significant GWAS associations in the locus of *Snn1* on chromosome 1BS for responses to all five *P. nodorum* isolates (SnToxA present in all isolates) in this study.

Our findings suggest the involvement of previously unidentified NEs in *P. nodorum*, as well as the presence of uncharacterized sensitivity/resistance genes in wheat. This was evident based on the GWAS, where multiple unknown loci were identified for responses to the five isolates and NEs (except ToxA). Of these, a locus on chromosome 2AS, *Qsnb.osu-2AS*, was found to be associated with responses to all five isolates and had greater effects than the *Tsn1* gene for some isolates. Phan et al. (2016) identified two QTL associated with response to SNB (*Qsnb.cur-2AS1* and *Qsnb.cur–2AS2*), which were mapped using Simple Sequence Repeat (SSR) and Diversity Arrays Technology (DArT) markers. We could not find the physical positions of the markers flanking *Qsnb.cur-2AS1*, whereas the physical position of *Qsnb.osu-2AS* (2.4–5.4 Mb on the Wheat Chinese Spring IWGSC RefSeq v2.1) was within the region of *Qsnb.osu-2AS.* Further work is needed to characterize the SNB resistance/susceptibility gene in *Qsnb.osu-2AS* and to understand its interaction with effector (s) in *P. nodorum*. The resistant allele of *Qsnb.osu-2AS* was present in about half of the germplasm, and the two developed KASP markers could be used to select for resistance.

The HWW variety ‘Green Hamme,’ which is currently the second most grown variety in Oklahoma (USDA Nass 2025), was insensitive to all five NEs and did not carry any of the corresponding sensitivity genes but was susceptible to isolates OKG16Sn1-1 and OKG16Sn-2. This suggests that other unreported sensitivity genes targeted by known or novel NEs could be present in Green Hammer and that other NEs could be identified in OKG16Sn1-1 and OKG16Sn-2. Mapping the susceptibility gene in Green Hammer to isolates OKG16Sn1-1 and OKG16Sn-2 is ongoing.

In conclusion, this study identified known sensitivity genes and novel loci associated with responses to *P. nodorum* isolates and NEs. This suggests that other NEs and resistance/sensitivity SNB genes could be identified in the *P. nodorum–*wheat system. As *Tsn1-B1* and *Snn3-B1/B2* are present in high frequencies in contemporary HWW, their elimination should be prioritized in HWW breeding programs using available KASP markers. This study provided four additional KASP markers linked to *Snn5-B1*, *Snn2*, and *Qsnb.osu-2AS*. This study focused on identifying the genetic factors underlying SNB susceptibility/resistance at the seedling stage. Future research is needed to identify loci associated with SNB response at the adult plant stage under field conditions.

## Supplementary Information

Below is the link to the electronic supplementary material.

Supplementary file1 (DOCX 7742 KB)

## Data Availability

All data generated analyzed during this study are included in this published article and its supplementary information files submitted with this manuscript. Supplementary Tables (Tables S1–S8) and Supplementary Figures (Figures S1–S10) are available at https://doi.org/10.6084/m9.figshare.33086756. The raw sequencing data of the wheat genotypes can be accessed from the NCBI Short-Read Archive BioProject PRJNA1468106 (https://www.ncbi.nlm.nih.gov/sra/PRJNA1468106).
